# Natural (∆^9^-THC) and synthetic (JWH-018) cannabinoids induce seizures by acting through the cannabinoid CB_1_ receptor

**DOI:** 10.1038/s41598-017-10447-2

**Published:** 2017-09-05

**Authors:** Olga Malyshevskaya, Kosuke Aritake, Mahesh K. Kaushik, Nahoko Uchiyama, Yoan Cherasse, Ruri Kikura-Hanajiri, Yoshihiro Urade

**Affiliations:** 1 International Institute for Integrative Sleep Medicine (WPI-IIIS), University of Tsukuba, Tennodai 1-1-1, Tsukuba, 305-8575 Japan; 2 Division of Pharmacognosy, Phytochemistry, and Narcotics, National Institute of Health Sciences, 1-18-1 Kamiyoga, Setagaya-ku, Tokyo 158-8501 Japan

## Abstract

Natural cannabinoids and their synthetic substitutes are the most widely used recreational drugs. Numerous clinical cases describe acute toxic symptoms and neurological consequences following inhalation of the mixture of synthetic cannabinoids known as “Spice.” Here we report that an intraperitoneal administration of the natural cannabinoid Δ^9^-tetrahydrocannabinol (10 mg/kg), one of the main constituent of marijuana, or the synthetic cannabinoid JWH-018 (2.5 mg/kg) triggered electrographic seizures in mice, recorded by electroencephalography and videography. Administration of JWH-018 (1.5, 2.5 and 5 mg/kg) increased seizure spikes dose-dependently. Pretreatment of mice with AM-251 (5 mg/kg), a cannabinoid receptor 1-selective antagonist, completely prevented cannabinoid-induced seizures. These data imply that abuse of cannabinoids can be dangerous and represents an emerging public health threat. Additionally, our data strongly suggest that AM-251 could be used as a crucial prophylactic therapy for cannabinoid-induced seizures or similar life-threatening conditions.

## Introduction

Marijuana (*Cannabis sativa L*.) is the most commonly abused drug in the United States^1^, with a similar tendency worldwide^2^. In the last few years its use for recreational and medical purposes has become legal in an increasing number of countries^3^. Particularly, in the United States marijuana use is illegal under federal law, nevertheless, it has been legalized by individual state laws for non-medical use in 8 states and is allowed for medical use by another 29 states^4^. A primary component of marijuana, Δ^9^-tetrahydrocannabinol (∆^9^-THC)^5^, mediates a psychoactive effect on the nervous system *via* cannabinoid receptor 1 (CB_1_R), for which it has a low binding affinity (K_i_ = 39.5 nM)^6^. However, in recent years, strains of herbal cannabis with an increased CB_1_R potency have appeared: one example is sinsemilla, a female non-pollinated cannabis plant in which the content of ∆^9^-THC has been raised from 3.96% in 1995 to 12.3% in 2014^1^. This recent shift in the generation of high-potency cannabis plant material has led to an increasing demand for cannabis-related treatments and emergency department admissions stemming from acute anxiety, psychosis or cognitive impairment^7–9^.

The advent of synthetic cannabinoids (SCs), a type of new recreational drug commonly known on the market as “Spice” created additional challenges. Since 2008 SCs have led to increased public awareness as the most rapidly growing group of new “legal highs”, with the ability to escape detection by standard cannabinoid screening tests. Once some substances have become regulated, novel analogues have emerged on the market to satisfy demand; and the speed of production is outpacing lawmakers’ attempts to ban them^10^. Most SCs act as full agonists at CB_1_R, with a much higher binding affinity (JWH-018, K_i_ = 9 nM; CP 47,497, K_i_ = 2.2 nM; HU210, Ki = 0.06 nM) compared to ∆^9^-THC^6, 11^. SCs can not only permeate the blood–brain barrier, but they also accumulate in CB_1_R-rich areas of the brain^12, 13^. Recently, the number of users displaying pathological behaviors after consuming SCs has dramatically increased; symptoms include anxiety, agitation, tachycardia, cardiotoxicity and seizures or status epilepticus^14, 15^. Deaths after SC use have also been documented: SCs were reported in 2014 to have killed 25 people and sickened more than 700 in northern Russia^16^. Controlled studies on humans to examine the action of cannabinoid ligands are difficult, and human data on induction, pharmacokinetics and adverse effects are therefore limited to case studies on users after voluntary drug consumption^17, 18^. At the same time, rapid analogue development and the growing popularity of SCs impose a strong demand for evaluation of their pharmacology and toxicology to reveal the mechanism of action and to facilitate future development of a drug-specific therapy for intoxication.

Here, we report, for the first time, that acute administration of a natural (∆^9^-THC, 10 mg/kg) or synthetic (JWH-018, 2.5 mg/kg) cannabinoid triggered electrographic seizures in mice, recorded by electroencephalography (EEG) and videography. We further show that cannabinoid-induced seizures were completely prevented by pretreatment of the animals with AM-251 (5 mg/kg), a CB_1_R-selective antagonist. Our data imply that even single use of cannabinoids may result in significant adverse neurological and physical effects and negatively affect human health. Finally, based on our results, AM-251 has a strong therapeutic potential for the suppression of toxic symptoms induced by cannabinoid abuse, although the human safety need to be established in controlled clinical trials.

## Results

### Cannabinoids induce electrographic seizures

**Figure 1:**
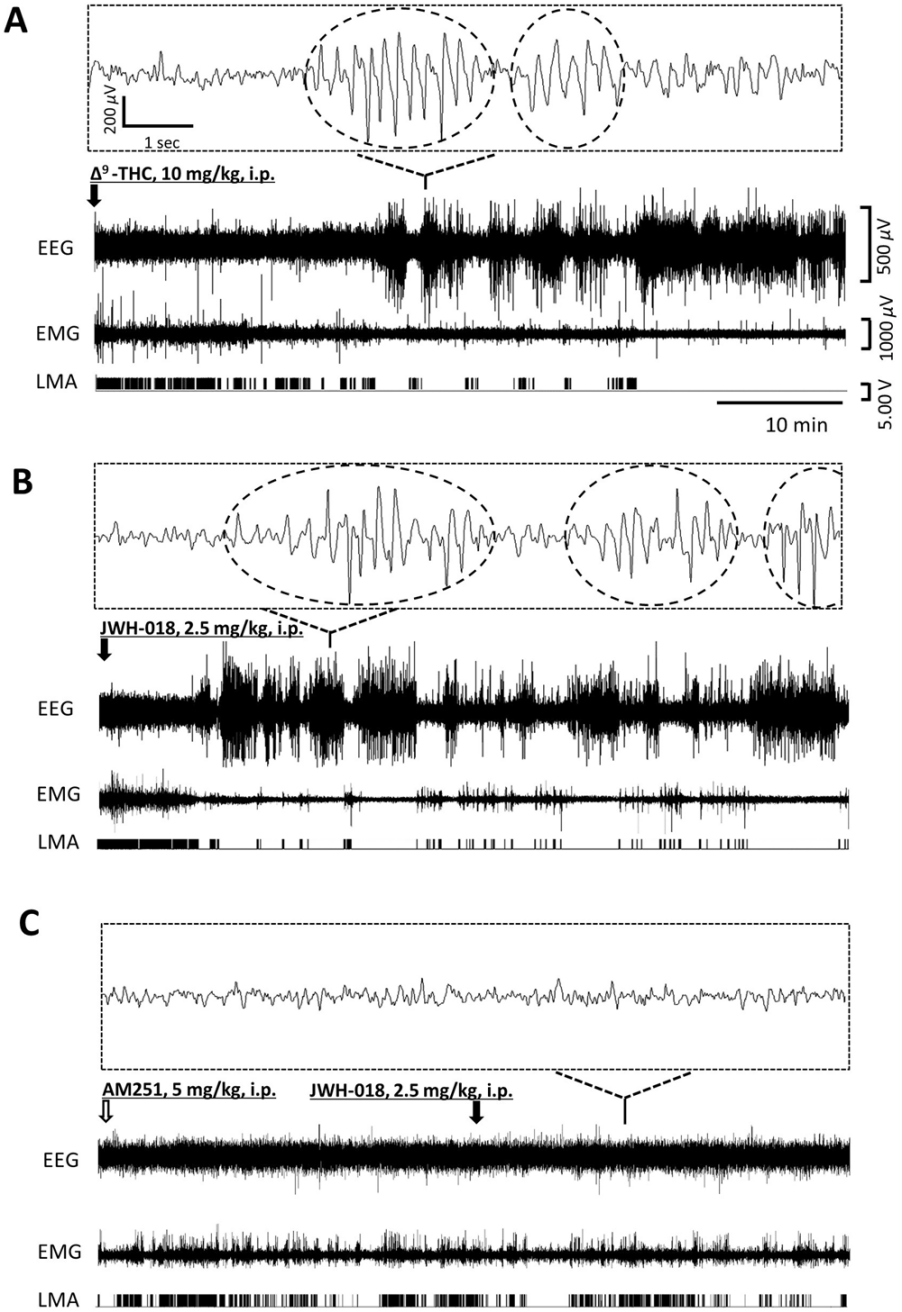
Effect of cannabinoids in mice. The arrow indicates the time of i.p. injection. (**A**) Injection of ∆^9^-THC (10 mg/kg) resulted in a high-amplitude EEG shortly after administration. In a magnified view, high-amplitude EEG seizure spikes (in dashed circles) were detected. EMG and LMA were decreased during this period. (**B**) Injection of the synthetic cannabinoid JWH-018 (2.5 mg/kg) also resulted in high-amplitude EEG and produced seizures, while EMG and LMA were low. (**C**) Pretreatment with the CB_1_R-specific antagonist AM-251 (5 mg/kg) 30 min before JWH-018 (2.5 mg/kg) prevented EEG seizures and EMG/LMA suppression. (**A**–**C**) n = 6.

**Figure 2:**
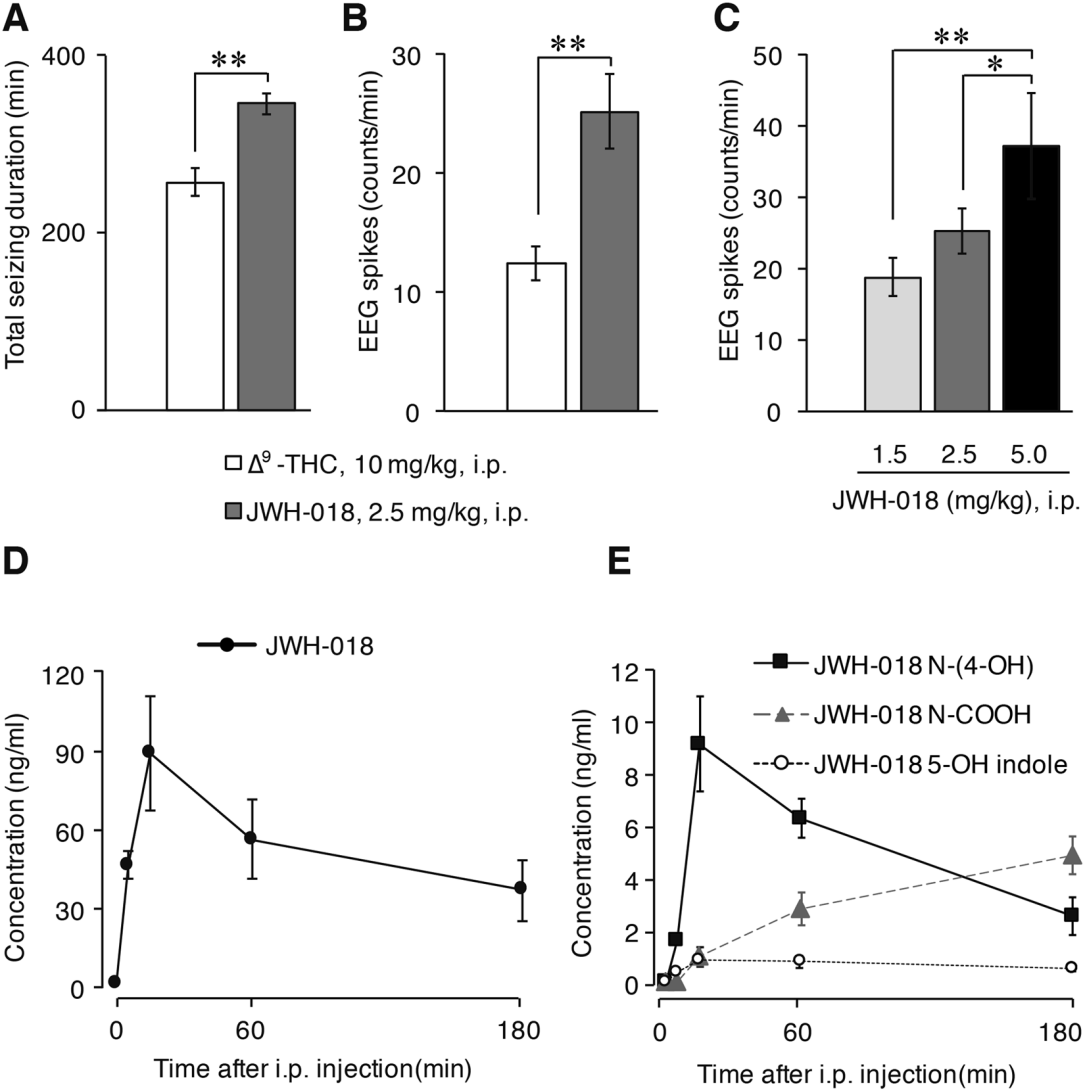
Characterization of cannabinoid-induced seizures and JWH-018 pharmacokinetics. (**A**) Total seizing duration (i.e., the period during which electrographic seizure spikes were apparent) for ∆^9^-THC (10 mg/kg) and JWH-018 (2.5 mg/kg) (**p = 0.0003). (**B**) EEG spike frequency for ∆^9^-THC, 10 mg/kg and JWH-018, 2.5 mg/kg (**p = 0.003). In (**A**,**B**) spike threshold 40–50%; only high-amplitude spikes were included in the analysis. Group comparison was done using the unpaired *t*-test. (**C**) JWH-018 administration induced seizure EEG in a dose-dependent manner (**p = 0.008; *p = 0.044, assessed by one-way ANOVA and LSD post-hoc analysis). The period analysed in (**B**,**C**) was from 0 to 2 h after i.p. administration of ∆^9^-THC or JWH-018. (**D**,**E**) Pharmacokinetics of JWH-018 (**D**) and its main metabolites (**E**) in serum. (**A**–**C**) n = 6, (**D**,**E**) n = 3.

We administered ∆^9^-THC (10 mg/kg) or JWH-018 (2.5 mg/kg), intraperitoneally to two independent groups of mice and recorded video, EEG, electromyogram (EMG) and locomotor activity (LMA). Shortly after administration, we observed a significant decrease in LMA and EMG activity that coincided with low-intensity behavioral seizures. Several minutes after cannabinoid administration, the EEG trace resembled non-rapid eye movement sleep (Fig. 1A,B). However, detailed inspection of a magnified view (Fig. 1A,B inset) revealed the presence of electrographic seizures in the form of frequent EEG seizure spikes. The average onset latency of electrographic/behavioral seizures was shorter after JWH-018 (5.4 ± 0.6 min; p < 0.05) compared to ∆^9^-THC administration (11.1 ± 2.5 min) (Fig. 1A,B). Electrographic seizures were apparent for 256 ± 15.3 minutes after ∆^9^-THC; however, after JWH-018 administration they persisted for longer (344 ± 12 min; p < 0.001) and were observed in all tested animals (Fig. 2A). Seizure spike quantification analysis revealed significantly more frequent spikes after JWH-018 (25.1 ± 3.1 spikes/min; p < 0.001) compared to ∆^9^-THC administration (12.3 ± 1.4 spikes/min) (Fig. 2B). Isolated EEG seizure spikes were still observed in both groups 4 h after administration (Suppl. Figure 1A,C); on the next day, the effect was diminished and no electrographic seizures could be detected (Suppl. Figure 1B,D).

We also observed behavioral changes after administration of JWH-018, including suppression of locomotor activity, impaired walking, ataxia, extensor rigidity in both hind limbs, straub tail, muscular jerks, rearing, and low-intensity behavioral seizures (Suppl. Video S1). The righting reflex was preserved, whereas the touch escape response was impaired. Animals also showed dyspnea (gasps) and profound catatonia (Suppl. Video S2). Similar behavioral changes were also observed after ∆^9^-THC administration; however, their intensity was lower than in the group administered JWH-018 (Suppl. Video S3). The maximal severity score reached under both JWH-018 and ∆^9^-THC was 3. The occurrence of events of score 2 or higher was more frequent for JWH-018 compared to ∆^9^-THC (Suppl. Table 1).

### Administration of JWH-018 elicits seizure spikes in a dose-dependent manner

To clarify whether cannabinoids induce seizure activity in a dose-dependent manner, we quantified spike frequency after acute i.p. administration of three different doses of JWH-018: 1.5, 2.5 and 5 mg/kg. The result showed a clear dose-response relationship, with the number of spikes increasing with dose (18.7 ± 2.7, 25.1 ± 3.1 and 37.1 ± 7.3 spikes/min respectively, p < 0.01 (1.5 vs. 5 mg/kg) and p < 0.05 (2.5 vs. 5 mg/kg), one-way ANOVA) (Fig. 2C).

### CB_1_R-selective antagonist AM-251 prevents cannabinoid-induced seizures

To determine whether the proconvulsive properties of cannabinoids were mediated by CB_1_R, we pretreated mice with AM-251 (5 mg/kg) 30 min before administration of JWH-018 (2.5 mg/kg). Pretreatment with AM-251 resulted in the complete abolition of electrographic seizures: the EEG showed a normal pattern in all animals tested (Fig. 1C). Moreover, behavioral abnormalities previously induced by administration of JWH-018 or ∆^9^-THC were absent in these AM-251-pretreated animals. Administration of AM-251 alone produced no detectable changes in the EEG or behavior of the animals (Suppl. Figure 2), however no specific behavioral tests, beyond simple observation were conducted.

### Metabolism of JWH-018 in serum

To examine the pharmacokinetics of JWH-018, blood was collected at different time points after administration of the drug at a dose of 2.5 mg/kg. After separation of the serum, JWH-018 and 6 metabolites were measured. In the samples, JWH-018 and JWH-018 N-(4-hydroxypentyl), [JWH-018 N-(4-OH)], JWH-018 N-pentanoic acid metabolites [JWH-018 N-COOH], JWH-018 5-hydroxyindole [JWH-018 5-OH indole] metabolites were detected (Fig. 2D,E). Serum JWH-018 concentration reached 45.5 ng/ml 5 min after administration, with a peak serum concentration of 87.8 ng/ml at 15 min post-injection. After 1 h the serum concentration still remained at 55.2 ng/ml and after 3 h the level of JWH-018 persisted at 35.7 ng/ml, which was still >40% of the peak concentration. JWH-018 N-(4-OH) metabolite increased in the serum concentration with a time profile similar to JWH-018, reaching its peak of 9.1 ng/ml at 15 min and slowly decaying to 6.4 ng/ml after 1 h and at 2.5 ng/ml at 3 h. Interestingly, the JWH-018-N-pentanoic acid metabolite (JWH-018 N-COOH) displayed a much slower increase in concentration, with the highest measured concentration of 4.9 ng/ml detected at 3 h after JWH-018 administration. JWH-018 5-OH indole metabolite increased in the serum at a much lower range of concentrations, with a peak at 0.8 ng/ml 15 min after administration. Other metabolites of JWH-018 were detected at low serum levels, with the highest value less than 0.9 ng/ml (data not shown).

## Discussion

Analysis of highly synchronized EEG (seizure spikes) and behavioral video recordings provide strong evidence for the epileptogenic properties of both synthetic (JWH-018) as well as plant-derived (∆^9^-THC) cannabinoids. Consistent with our data, a previous study has shown that injections of ∆^9^-THC in rats induce burst activity in the EEG^19^; and even a single dose of ∆^9^-THC can promote a long-lasting elevation of neuronal excitability, thus increasing susceptibility to seizure^20^. On the other hand, a substantial body of literature on cannabinoids in animal models shows mostly anticonvulsive effects^21^. However, few of these used EEG recordings to assess epileptic events and many of them induced seizures either electrically or pharmacologically, changing signalling pathways and brain states prior to cannabinoid application.

Since the discovery of the cannabinoid system (CB_1_R, CB_2_R and endogenous cannabinoids), their physiological properties have been extensively discussed and contradictory findings have been reported in humans as well. Some studies suggest that cannabinoids exert severe side effects along with strong proconvulsive properties^22, 23^, whereas others have shown anticonvulsive effects and used cannabinoids as a “relief drug” for severe epilepsy cases^24, 25^. Despite such attempts to use medical marijuana in the treatment of intractable epilepsy, the medical evidence supporting its use for the treatment of neurological diseases is not sufficiently convincing^26^, and evidence for the safety and efficacy of cannabinoids that would allow them to be used clinically remains weak. It is also important to note that in these human studies ∆^9^-THC was rarely used as a pure chemical, but as a part of marijuana plants or medical preparations, containing cannabidiol, another component of *Cannabis sativa*, that exerts major antiepileptic effects^27^.

Because we intentionally used higher doses which produce toxicity and induce seizures, these doses might have effects not representative of those seen with typical medicinal or recreational human consumption. It would be interesting in the future to also test lower doses, typically used medicinally or recreationally to determine whether the effect is lost or diminished. As we show here, seizures induced by cannabinoids are typically mild and almost asymptomatic to the untrained eye - therefore careful EEG evaluation would be needed. The highest reported concentration of JWH-018 in our study was 87 ng/ml at 15 min after administration, which is about 9 to 17 times higher than the value published in a controlled human studies after inhalation^18, 28^, where doses are far away from toxic ones for humane and safety reasons. After 3 h serum concentration reached 36 ng/ml which is three times higher than previously reported for intoxications in humans^29^. However, it is important to note that dose conversion always requires some consideration, because human bioavailability and metabolism may differ significantly from that of animals. Furthermore, differences between human and animal receptor sensitivity or density have been shown to affect human pharmacologic or toxicologic outcomes^30^. In general, number of studies on JWH-018 pharmacokinetics are limited and sample sizes with equal conditions are small, which makes the comparison of the different studies difficult. Another point is the different route of administration, and it is possible that inhalation might have a faster pharmacokinetic effect than intraperitoneal injection.

The activity of metabolites of JWH-018 is unknown, but may be important because such metabolites may exert synergistic effects and reinforce the SCs toxicity. Among them, the JWH-018 N-(4-OH) metabolite exhibited the highest concentration and mimicked the time/concentration curve of JWH-018. Interestingly, in humans, the JWH-018-N-pentanoic acid metabolite usually demonstrates the highest concentration^28^ among metabolites, while our data showed the concentration slowly beginning to rise after 15 min. This may reflect a species difference for JWH-018 metabolism, and thus potentially indicate different toxicity. This increase of concentration could partially explain the long-term electrographic and behavioral changes after JWH-018 administration in mice. To answer these questions, further studies on JWH-018 metabolites and their toxicity need to be conducted in mice.

Both ∆^9^-THC and JWH-018 induced breathing alterations and profound catatonia, which are consistent with previous reports^31^. At the same time, JWH-018 produced severe adverse neurological effects with durations longer than ∆^9^-THC, including extensor hind limb rigidity, muscular jerks, rearing, and behavioral seizures; this longer duration and severity may be attributable to differences in blood-brain permeability or intracerebral distribution of these compounds^12, 13^. CB_1_Rs are expressed in both excitatory glutamatergic and inhibitory GABAergic terminals; therefore, the distribution of CB_1_R-expressing activated neurons may play a key role in generating different behavioral and neurophysiological effects^32^.

Our results clearly show that the epileptogenic properties of cannabinoids are mediated via CB_1_ receptors. Therefore, pure CB_1_R agonism, or high affinity to CB_1_Rs carries a high risk to induce seizures. The greatest danger therefore for accidental overdoses seems to stem from SCs with high CB_1_R affinity. These substances are often not controlled, since they are newly synthesized and therefore not yet banned by regulatory agencies. This may lead users to falsely believe that they are safe for consumption. Combined with unknown doses and affinities, users may easily subject themselves to accidental overdose. Patients that present to emergency rooms with a history of SC abuse should be carefully monitored for epileptic events using EEG recordings. Particularly since their symptoms might be misinterpreted as simple sedation, stupor or catatonia^33, 34^. Use of plant-derived cannabinoids seems less risky, since their potency might be lower and anti-epileptogenic compounds may counterbalance the epilepsy risk posed by CB_1_R agonism. Nevertheless these cannabinoids, if overdosed, can lead to epileptic seizures, as we show here. Thus, the same vigilance as for SCs seems prudent, if overdose is suspected. Controlled medical use of cannabinoids seems to carry the smallest risk. Doses are typically significantly lower than used in our studies to elicit seizures. It should be noted, however that epileptic seizures as rare adverse side effects of medical marijuana use have been reported^35^.

The CB_1_R agonists we have studied revealed strong proconvulsive properties, implying that any newly synthesized CB_1_R agonists may also exert similar behavioral effects and trigger seizures. Seizures require immediate treatment because they can be life-threatening and/or disabling. No specific medication is currently available to alleviate cannabinoid intoxication. The mechanism of toxicity to explain the seizures induced by cannabinoids remains unclear. Coupled with increased public use, constant production of new analogues and our lack of knowledge as to their long-term effects on human health show the need for further research and proper regulation. Finally, the potential introduction of CB_1_R antagonists, as a treatment for cannabinoid-induced seizures or other life-threatening conditions in the case of overdose, requires further investigation in the clinical settings.

## Methods

### Animals

All experiments were conducted using adult male C57BL/6J mice at the age of 10–14 weeks, obtained from Charles River (Kanagawa, Japan). All described experiments were replicated at least twice. Mice were housed in a temperature- and humidity-controlled environment and maintained on a 12-h light: 12-h dark cycle (lights on at 07:00). Food and water were available *ad libitum*. Experiments were performed in compliance with relevant Japanese and institutional laws and guidelines and approved by the Animal Ethics Committee of the University of Tsukuba, number 16–086. Efforts were made to reduce the number of animals used and to minimize any pain or discomfort they might have felt.

### EEG/EMG implantation surgery

Under anaesthesia with pentobarbital (50 mg/kg, i.p.), mice were placed on an animal stereotaxic frame (David Kopf Instruments, Tujunga, CA, USA), after which custom-made EEG and EMG electrodes were implanted aseptically. EEG electrode screws were placed epidurally on the right frontal and parietal cortices (AP, +1 mm to the front of bregma or lambda; LR −1.5 mm lateral from the sagittal raphe) according to the mouse brain atlas of Franklin and Paxinos^36^. EMG signals were recorded from two insulated Teflon-coated electrodes that had been inserted into the bilateral neck muscles. Finally, the electrode assembly was fixed to the skull with self-curing dental cement, and the wound was then sutured. After surgery, the animals were administered an i.p. injection of ampicillin (100 mg/kg) and allowed to recover in individual housing cages for one week. Each animal was then connected to an EEG/EMG recording cable, with acclimation in an individual sound-proof recording chamber for an additional three days. Each cable was flexible so that the mice could move freely about their cages.

### Video, EEG, EMG and LMA polygraphic recording and analysis

Cortical EEG and EMG signals were amplified, filtered (EEG, 0.5–35 Hz; EMG, 20–200 Hz) and digitized at a 128-Hz sampling rate. The signals were recorded by using Vital Recorder software (Kissei Comtec, Nagano, Japan) and analysed with Sleepsign software (Kissei Comtec) as previously described^37^. A passive infrared sensor was placed on each animal’s cage to detect movement of the subject. A video of each animal was recorded using an infrared camera.

### Video behavioral assessment and quantification

To characterize each animal’s condition and behavior more precisely, we performed video recording together with live EEG monitoring using a video camera (Panasonic HC-WX990 M) in the double-screen mode. Severity of typical seizure symptoms was assessed by Racine’s scale^38^ with modifications: 0 - no behavioral change; 1 - immobility and behavioral arrest; 2 - isolated body jerks; 3 - rearing and tonic extension of forelimbs and hind limbs, occasional clonus.

### Seizure-spike quantification

Seizure spikes were detected and counted using peak analysis function of OriginLab v8.5 Pro software (OriginLab, Northampton, MA, USA). A threshold from 40 to 50% was set to restrict quantification to high-amplitude spikes only.

### Drugs

Δ^9^-THC was provided by Dr. S. Morimoto (Pharmaceutical Sciences, Kyushu University, Fukuoka, Japan) and stored in ethanol at −80 °C. JWH-018, metabolites of JWH-018, deuterium-labelled JWH-018 (JWH-018-d9) and AM-251 were purchased from Cayman Chemical (Ann Arbor, MI, USA) and stored at −20 °C. The doses of cannabinoids presently used here were selected based on the CB_1_R affinity of the ligands and the electrographic representation of mild seizure events. In fact, we intentionally selected relatively high doses that we knew to have an effect on EEG power spectra^39^. The conversion of an animal to human dose cannot be done directly, as in the few available studies on human volunteers most utilize the inhalation route, while we administered drugs intraperitoneally. However, after applying the human estimation dose conversion^40^, 2.5 mg/kg of JWH-018 in mouse corresponds to 0.2 mg/kg, or roughly 12 mg per person. Similarly, 10 mg/kg of ∆^9^-THC in mice converts to 0.8 mg/kg in humans or about 49 mg per individual.

### Drug administration

All chemicals were dissolved in a mixture of 5% dimethylsulfoxide, 5% emulphor (EL-620, a polyoxyethylated vegetable oil; GAF Corporation, Linden, NJ, USA), and 90% saline (0.9% NaCl). Drug solutions were prepared immediately before use and administered intraperitoneally 1 h after dark onset. Each animal received a vehicle injection, which served as a control; the cannabinoid (Δ^9^-THC or JWH-018) was then injected the following day. AM251 was injected 30 min before JWH-018 administration.

### Measurement of serum JWH-018 and metabolites using liquid chromatography–coupled to tandem mass spectrometry (LC-MS/MS)

Blood was collected in Terumo Capiject Capillary Blood Collection Tubes (Tokyo, Japan) and, after centrifugation serum was transferred into plastic tubes. JWH-018-d9 was added as an internal standard (IS) to each serum sample, and the samples were filtrated using Captiva ND^Lipids^ (Agilent, Santa Clara, CA, USA). Partially purified samples were injected into the I-Class/Xebo TQS LC–MS/MS system (Waters, Milford, MA, USA) equipped with an electrospray interface and operated in the positive ion mode. A Coretecs C18 column (2.7 μm, 2.1 mm i.d. ×150 mm; Waters) together with a mobile phase consisting of 0.1% (v/v) formic acid/water (solvent A) and 0.1% formic acid/acetonitrile (solvent B) was used for LC separation. Quantification of JWH-018 and six metabolites was performed using multiple reaction monitoring (MRM) of the transitions of precursor ions to product ions with each cone voltage and collision energy as shown in Table 1. During the analyses, we confirmed two transitions (precursor ions and two product ions of each compound) and their ratio. The drug concentrations in the samples were calculated using the peak–area ratios of the product ions for quantitative monitored for the target compounds versus IS. The calibration curves for the determination were constructed by analysing extracted drug-free control serum spiked with the standard solution. The limit of quantitation of each drug was chosen to be the concentration of the lowest calibration standard with an acceptable limit of variance, while the limit of detection was defined as concentration in a sample matrix resulting in peak areas with signal-to-noise ratios (S/N) of 3. Calibration curves of JWH-018 and the metabolites were linear over the concentration range 0.2–400 ng/ml for JWH-018 and 0.2–40 ng/ml for the metabolites with good correlation coefficients of r^2^ ≥ 0.995, respectively. The limit of detection of each drug was 0.02 ng/ml.

**Table 1:** MRM conditions used for quantitative analysis of JWH-018 and its metabolites.

	Retention time (min)	MRM conditions		Cone voltage (V)	Collision voltage (eV)
		Precursor ions	Product ions		
JWH-018 N-pentanoic acid metabolite [JWH-018 N-COOH]	9.22	372	155	20	25
JWH-018 N-(5-hydroxypentyl) metabolite [JWH-018 N-(5-OH)]	9.62	358	155	14	21
JWH-018 N-(4-hydroxypentyl) metabolite [JWH-018 N-(4-OH)]	9.79	358	155	14	21
JWH-018 N-(3-hydroxypentyl) metabolite [JWH-018 N-(3-OH)]	11.21	358	155	14	21
JWH-018 6-hydroxyindole metabolite [JWH-018 6-OH indole]	13.08	358	155	20	21
JWH-018 5-hydroxyindole metabolite [JWH-018 5-OH indole]	13.37	358	155	20	21
JWH-018	18.67	342	155	28	23
JWH-018-d9 (IS)	18.54	351	155	36	24

### Group assignment

All animals were littermates and were assigned randomly to different groups. EEG analysis and spike quantification were performed by an experimenter who was unaware of the group to which the animal belonged.

### Sample size

Power analysis was used to determine the ideal sample size for behavioral experiments. Assuming a normal distribution, a 20% change in mean and 15% variation, we estimated that six mice would be required per group. In some cases, mice were excluded from analysis due to damage to or loss of the EEG electrode.

### Statistical analysis

Data are presented as individual mean ± s.e.m. for groups, where n = 6, with the exception of the metabolite analysis, where n = 3. Group comparison was done using the unpaired two-tailed *t*-test; and in all cases, *p* ≤ 0.05 was considered significant. For dose-dependent experiments one-way ANOVA followed by LSD post-hoc analysis was performed using SPSS 22.0 software (IBM SPSS Statistics, Armonk, NY, USA).

The data sets generated and analysed during the current study are available from the corresponding author on reasonable request.

## Electronic supplementary material

Video S1

Video S2

Video S3

Supplementary information
