# Supplementary material for: Natural (∆9-THC) and synthetic (JWH-018) cannabinoids induce seizures by acting through the cannabinoid CB1 receptor
Source: Sci Rep. 2017 Sep 5;7:10516. doi: 10.1038/s41598-017-10447-2 (PMC5585372; doi:10.1038/s41598-017-10447-2)
Supplement: Supplementary file 4 — Supplementary information [file 41598_2017_10447_MOESM4_ESM.pdf]

Title: Natural (Δ^9-THC) and synthetic (JWH-018) cannabinoids induce seizures by acting through the cannabinoid CB_1 receptor.

Authors

Olga Malyshevskaya[1*], Kosuke Aritake[1], Mahesh K. Kaushik[1], Nahoko Uchiyama[2], Yoan Cherasse[1], Ruri Kikura-Hanajiri[2], Yoshihiro Urade[1]

Supplementary Information

Suppl. Fig. 1. EEG and EMG traces after administration of cannabinoids. A. At 4 h after Δ9-THC (10 mg/kg) administration. **B.** At 24 h after Δ9-THC (10 mg/kg) administration. **C.** At 4 h after JWH-018 (2.5 mg/kg) administration. **D.** At 24 h after JWH-018 (2.5 mg/kg) administration. EEG spikes are circled in the enlarged view. The analysed period in **B** and **D** includes the first hour of the dark phase of the following day.

Suppl. Fig. 2. EEG, EMG and LMA traces following administration of AM-251 (5 mg/kg).

Video S1. Seizure activity following i.p. administration of synthetic cannabinoid JWH-018.

Video S2. Behavioral reactions to i.p. administration of synthetic cannabinoid JWH-018.

Video S3. Behavioral reactions to, and seizure activity after, i.p. administration of Δ9-THC.

Suppl. Table 1. Behavioral events duration after Δ^9-THC (10 mg/kg) or JWH-018 (2.5 mg/kg) administration. Events were quantified for a period of 1 h following i.p.; only

behavior with a score 2 and above (see Methods) were included in the analysis. (*, $p > 0.05$, unpaired t-test)

Suppl. Fig. 1

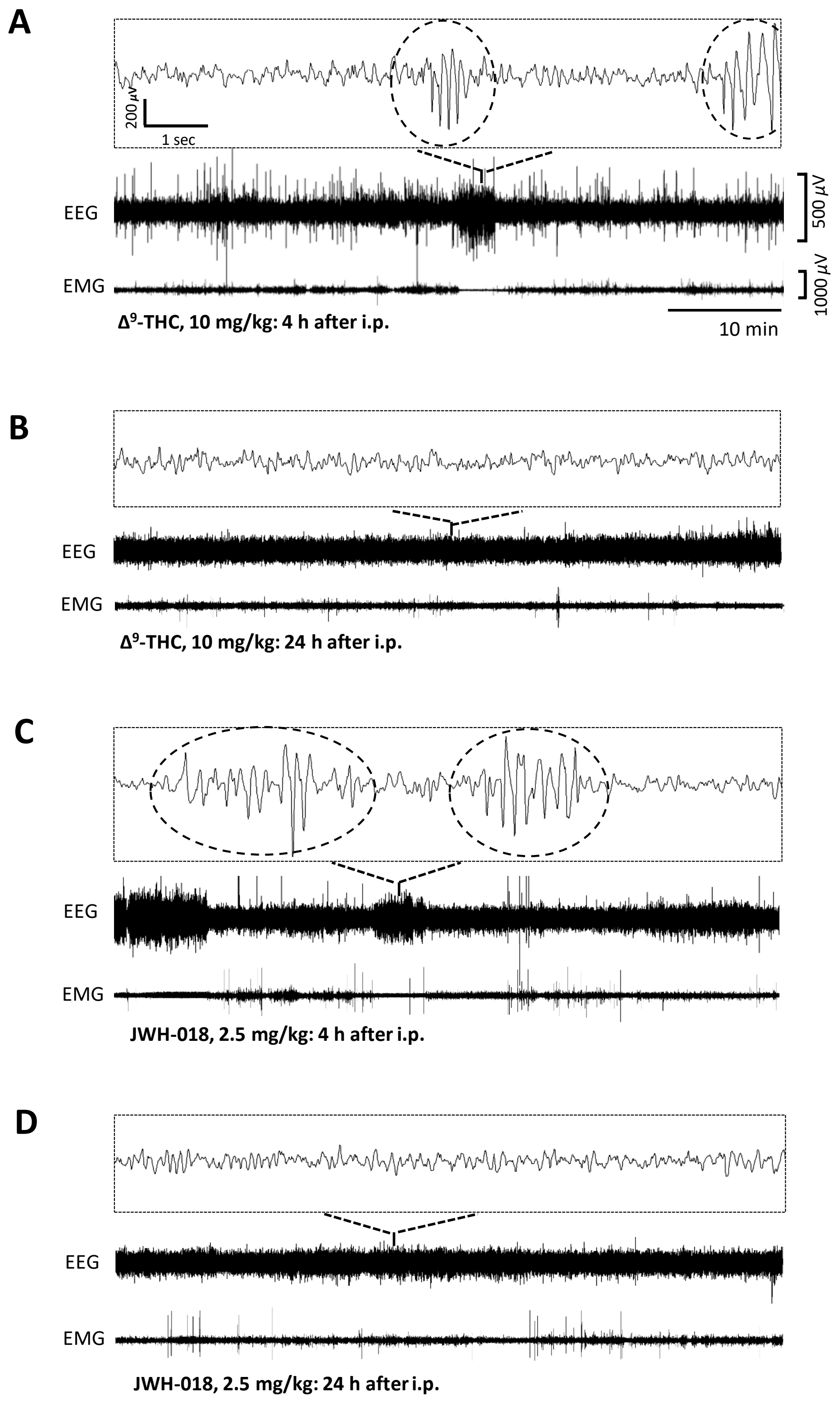
A
200 µV
1 sec
EEG
EMG
500 µV
1000 µV
10 min
Δ9-THC, 10 mg/kg: 4 h after i.p.
B
EEG
EMG
Δ9-THC, 10 mg/kg: 24 h after i.p.
C
EEG
EMG
JWH-018, 2.5 mg/kg: 4 h after i.p.
D
EEG
EMG
JWH-018, 2.5 mg/kg: 24 h after i.p.

Suppl. Fig. 2

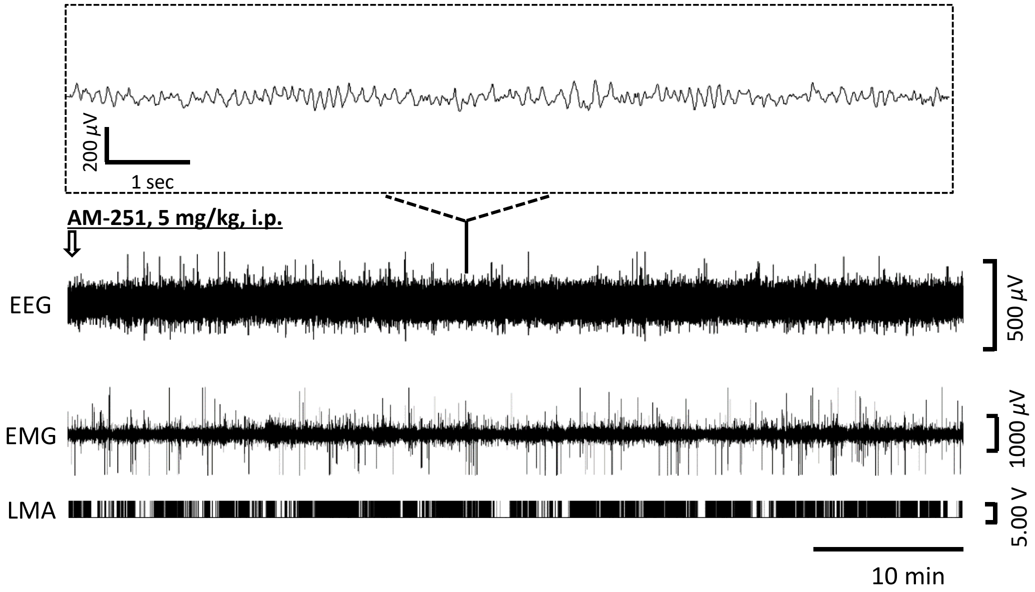

200 µV
1 sec
AM-251, 5 mg/kg, i.p.
EEG
500 µV
EMG
1000 µV
LMA
5.00 V
10 min

Suppl. Table 1

		Δ9-THC (10 mg/kg)	JWH-018 (2.5 mg/kg)
events/h			
average number (± s.e.m.)			
duration	1-4 sec	15.2 ± 4.3	34 ± 4.4*
	5-8 sec	0	6 ± 1*
	Total	15.2 ± 4.3	40 ± 4.5*
